# Characterization of complete chloroplast genome of *Ulva torta* (Mertens) Trevisan, 1841

**DOI:** 10.1080/23802359.2022.2081943

**Published:** 2022-06-14

**Authors:** Qinlin Wen, Weiming Yang, Jingshi Li, Jinlin Liu, Shuang Zhao, Song Gao, Jianheng Zhang, Peimin He

**Affiliations:** a College of Marine Ecology and Environment, Shanghai Ocean University, Shanghai, China; b Haiyan County Aquaculture Techniques Station of Zhejiang Province, Haiyan, China; c College of Marine Resources and Environment, Hebei Normal University of Science and Technology, Qinhuangdao Hebei, China; d North China Sea Marine Forecasting Center, State Oceanic Administration, Qingdao, China

**Keywords:** chloroplast genome, macroalgae, phylogenetic analysis, *Ulva torta*

## Abstract

*Ulva torta* (Mertens) Trevisan, 1841 was a global temperate widespread species. Green tide blooms caused by the green algae of the *Ulva* species occurred frequently in China. As a newly discovered species in the green tide bloom area, it was necessary to explore the relationship between *U.* torta and other green algae of the *Ulva* species. The complete chloroplast genome of *U.* torta was 105,423 bp in size. A total of 100 genes were annotated in the genome, containing 70 protein-coding genes, 27 transfer RNA (tRNA) genes, and three rRNA genes. The chloroplast genome had high AT content (74.76%). Phylogenetic analysis showed *U. torta* was clustered with *Ulva meridionalis*. This work could be useful for studying the evolution and genetic diversity of *U. torta*.

*Ulva torta* (Mertens) Trevisan, 1841 was widely distributed over most temperate sea area of the world (Guiry and Guiry 2021), mainly living in the waters of 20–25 °C (Ogawa et al. 2013). First report of *U. torta* was in Germany in 1822 (Silva et al. 1996). Thalli of *U. torta* were emerald green, slightly compressed or tubular, and consisted of a single layer of cells. Many branches were found at the base of the thallus, similar to the *Ulva clathrata* and *Ulva flexuosa* morphologies (Ogawa et al. 2013; An and Nam 2017). Chloroplast genome sequence analysis of more *Ulva species* could be a promising approach for further understanding the evolutionary history of this eukaryotic lineage.

*U. torta* (gametophyte) was collected from the sea area of Rudong, Jiangsu, China in November, 2020 (32°25′52″N, 121°24′35″E). The specimen was deposited at the herbarium of Shanghai Ocean University Museum (collected by Qinlin Wen, wenql587@163.com) under the voucher number SHOU2020RDA21121. The specimen was sent to Sangon Biotech (Shanghai) Co., Ltd. for DNA extracting and high-throughput sequencing. DNA was extracted from this sample using the company's Dzup (Plant) Genomic DNA Isolation Reagent. TruSeq DNA Sample Prep Kit was used to prepare genomic shotgun library (Illumina, USA), and then paired-end sequences were obtained by Illumina HiSeq 2500 platform. We obtained 10,626,405 raw read pairs and 1.59 Gbp data, with a single read length of 150 bp.

The chloroplast genome of *Ulva compressa* (MT916929) (Xia et al. 2021), *Ulva meridionalis* (MN889540) (Liu et al. 2020), *U. flexuosa* (KX579943) (Cai et al. 2017), *Ulva prolifera* (KX342867) (Jiang et al. 2019) and *Ulva linza* (KX058323) (Wang et al. 2017) had been studied by our laboratory before. Chloroplast genome of *U. prolifera* was taken as seed sequences for sequence splicing about the complete chloroplast genome of *U. torta* using NOVOPlasty software (Sedanza et al. 2020). The extended chloroplast genome contigs was obtained, and then the extended contigs were compared with the chloroplast genome sequence of *U. prolifera* to obtain the correct contig sequence. Then the complete chloroplast genome sequence was perfectly assembled.

Complete chloroplast genome of *U. torta* was 105,423 bp in size (GenBank accession number MZ703011). The chloroplast genome composition was biased toward AT content, at 74.76%, which was significantly higher than the GC content. When we annotated the whole chloroplast genome, *Ulva ohnoi* was mainly used for reference. A total of 100 functional genes were encoded in the genome, including 70 protein-coding genes, three rRNA genes (rrn 16 s gene, rrn 23 s gene, rrn 5 s gene, respectively), and 27 tRNA genes. In order to verify the phylogenetic position of the newly obtained species within *Ulva* species and further clarify the evolutionary relationship, phylogenetic analysis was carried out with 12 species (*Pseudendoclonium akinetum* as an outgroup taxonomically belongs to Chlorophyta, Ulvophyceae, Ulvales, Kornmanniaceae, *Pseudendoclonium*). All chloroplast genomes sequences were aligned with the BioEdit sequence software (Hall 1999). Clustal X software was used to perform multiple alignment analysis on qualified sequences. A Maximum Likelihood (ML) tree was constructed by Mega 7.0 (Kumar et al. 2016), and the accuracy of the phylogenetic tree was verified by the Bootstrap test repeated 1000 times, omitting less than 50% of the phylogenetic tree branch values. The result showed *U. torta* was related to *U. meridionalis* (Figure 1).

**Figure 1.**
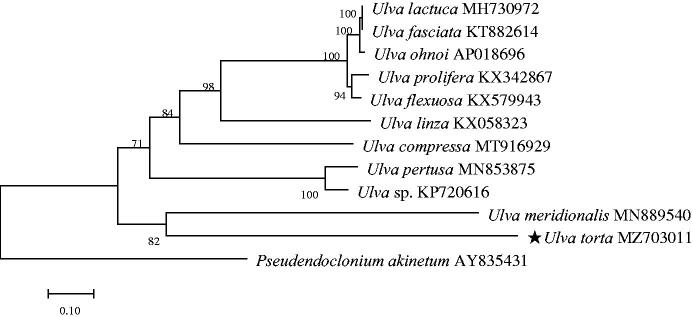
A ML phylogenetic tree for *U. torta* based on the whole chloroplast genomes of 10 other closely related species (*U. compressa* is 94,226 bp (MT916929) (Xia et al. 2021), *U. meridionalis* is 88,653 bp (MN889540) (Liu et al. 2020), *U. flexuosa* is 89,414 bp (KX579943) (Cai et al. 2017), *U. prolifera* is 93,066 bp (KX342867) (Jiang et al. 2019), *U. linza* is 1,251 bp (KX058323) (Wang et al. 2017), *U. ohnoi* is 103,313 bp (AP018696) (Suzuki et al. 2018), *Ulva* sp. is 99,983 bp (KP720616) (Melton et al. 2015), *Ulva fasciata* is 96,005 bp (KT882614) (Melton and Lopez-Bautista 2016), *Ulva lactuca* is 95,997 bp (MH730972) (Hughey et al. 2019) and *Ulva pertusa* is 104,380 bp (MN853875) (Han et al. 2020)) and one outgroup (*Pseudendoclonium akinetum* is 195,867 bp (AY835431) (Pombert et al. 2006)).

In this study, we analyzed complete chloroplast genome of *U. torta.* Currently, *Ulva* macroalgal blooms occur frequently in the Southern Yellow Sea of China (Zhang et al. 2014; Zhang et al. 2017; Zhao et al. 2019; Liu et al. 2020; Xiao et al. 2020; Liu et al. 2021). This study about chloroplast genomes of *Ulva* species will be useful for studying their genetic diversity.

## Data Availability

The support genome sequences data of this study are openly available in GenBank of NCBI (https://www.ncbi.nlm.nih.gov/) under the accession no. MZ703011. The associated BioProject, Bio-Sample, and SRA accession numbers are PRJNA768934, SAMN22561212, and SRR16629507, respectively.
